# DNA Methylation Supports Intrinsic Epigenetic Memory in Mammalian Cells

**DOI:** 10.1371/journal.pgen.0020065

**Published:** 2006-04-28

**Authors:** Yong-Qing Feng, Romain Desprat, Haiqing Fu, Emmanuel Olivier, Chii Mei Lin, Amanda Lobell, Shilpa N Gowda, Mirit I Aladjem, Eric E Bouhassira

**Affiliations:** 1 Department of Medicine, Division of Hematology, and Department of Cell Biology, Albert Einstein College Of Medicine, Bronx, New York, New York, United States of America; 2 Laboratory of Molecular Pharmacology, National Cancer Institute, Bethesda, Maryland, United States of America; The Babraham Institute, United Kingdom

## Abstract

We have investigated the role of DNA methylation in the initiation and maintenance of silenced chromatin in somatic mammalian cells. We found that a mutated transgene, in which all the CpG dinucleotides have been eliminated, underwent transcriptional silencing to the same extent as the unmodified transgene. These observations demonstrate that DNA methylation is not required for silencing. The silenced CpG-free transgene exhibited all the features of heterochromatin, including silencing of transcriptional activity, delayed DNA replication, lack of histone H3 and H4 acetylation, lack of H3-K4 methylation, and enrichment in tri-methyl-H3-K9. In contrast, when we tested for transgene reactivation using a Cre recombinase-mediated inversion assay, we observed a marked difference between a CpG-free and an unmodified transgene: the CpG-free transgene resumed transcription and did not exhibit markers of heterochromatin whereas the unmodified transgene remained silenced. These data indicate that methylation of CpG residues conferred epigenetic memory in this system. These results also suggest that replication delay, lack of histone H3 and H4 acetylation, H3-K4 methylation, and enrichment in tri-methyl-H3-K9 are not sufficient to confer epigenetic memory. We propose that DNA methylation within transgenes serves as an intrinsic epigenetic memory to permanently silence transgenes and prevent their reactivation.

## Synopsis

Regulation of gene expression during development and differentiation occurs at many levels including transcriptional gene silencing and activation. Understanding the mechanism of gene silencing has practical importance because genes inserted in the genome for gene therapy are often silenced, decreasing the efficiency and increasing the risk associated with these procedures. In this report, the authors created a gene that cannot be methylated by mutating all of its CpG dinucleotides and examined in detail the role of DNA methylation and other epigenetic modifications in the establishment and maintenance of gene silencing. The authors found that DNA methylation is not required for the establishment or the maintenance of the silent chromatin state, but that this covalent DNA modification was unique among the epigenetic marks tested because it conferred to the chromatin structure a long-term intrinsic epigenetic memory that prevents gene reactivation.

## Introduction

Silencing of transgenes, viruses, and transposons is a general phenomenon that has been documented from yeast to mammals [1] and is believed to be part of a genome defense mechanism against mobile genetic elements [2]. Understanding the epigenetic mechanisms underlying transgene silencing should provide insights into this important nuclear process and also have practical importance: for instance, designing safer gene therapy cassettes. Numerous studies have shown that gene expression in general, and transgene expression in particular, is regulated in part by DNA methylation, post-translational histone modifications, and timing of replication, three of the major epigenetic factors controlling chromatin structure.

DNA methylation is associated with transgene silencing, X chromosome inactivation, genomic imprinting, silencing of mobile genetic elements, and the developmental regulation of some tissue-specific genes [3]. In mammalian cells, DNA methylation is maintained and established by three DNA methyltransferases (Dnmt) [4]. Dnmt3a and Dnmt3b are particularly important for de novo methylation, while Dnmt1 is critical for the maintenance of DNA methylation after DNA replication [5]. The greater affinity of Dnmt1 for hemi-methylated DNA than for unmethylated DNA has been suggested as the mechanism for the maintenance of methylation patterns after each cell division (reviewed in [6]).

Histone modifications have emerged as major mediators of the formation of repressive chromatin structures [7]. Deacetylation of lysines in the tails of histone H3 and H4 is associated with silent chromatin, while acetylation of the same lysine residues is associated with gene activation [8,9]. Methylation of lysine 4 of histone H3 (H3-K4-Me) is associated with active chromatin, while methylation of H3-K9 and H3-K27 has been shown to be important for the formation of constitutive and facultative heterochromatin [10–14].

The maintenance of histone modifications after each DNA replication is not fully understood. Once certain silent chromatin structures are established, they can be stably transmitted through cell division in the absence of the initiating signal [15]. This epigenetic memory is best understood for genes regulated by the Polycomb proteins in *Drosophila* and by the Sir proteins in yeast [15–17]. A similar phenomenon has been elegantly demonstrated in mammalian cells by the Rauscher lab [10]. In that report, the memory was conferred to the transgenes by the recruitment of HP1.

Models for the inheritance of histone modifications are based on the observations that the effectors of silencing that bind post-translationally modified histones can also recruit the enzymes that modify the histones. After DNA replication, the newly assembled nucleosomes are believed to be made of a random mixture of new unmodified histones and of the old modified histones. Binding of effector proteins to the old histones would recruit histone-modifying enzymes and lead to the re-establishment of the pattern of histone modifications that existed before DNA replication [16,18].

In mammals, multiple histone-modifying enzymes and chromodomain proteins are known to be important for silencing, but the interactions between DNA methylation and histone modifications are complex. The Dnmts have been shown to interact with at least 15 proteins including HP1, histone methyltransferases, and histone deacetylases [19–21]. Whether histone modifications and DNA methylation are redundant, cooperative, or independent pathways of gene silencing remain unclear.

Timing of replication during S phase is likely to be an important factor for gene silencing since the epigenetic information must be replicated after each cell cycle [22]. This was suggested more than 15 years ago by the observation that repressed genes tend to replicate later in S phase than active genes. More recently, Simon et al. [23] provided support to the hypothesis that timing of replication affects the maintenance of epigenetic information by showing that different histone modifications were added to transgenes introduced early or late in S phase. Importantly, we recently demonstrated that the timing of replication of transgenes inserted into reference integration sites in mammalian chromosomes correlates with expression and the condensation state of chromatin, as manifested by histone modifications and methylation on CpG residues [24]. However, whether the timing of replication is a cause or a consequence of silencing, and whether it contributes to the maintenance of the silenced or active states, is still unclear.

In this study, we aimed to assess the relative contribution of CpG methylation, timing of replication, and histone modifications in the establishment and maintenance of transcriptionally silent, late replicating, and condensed chromatin domains. We found that silencing of transcriptional activity, delayed DNA replication, lack of histone H3 and H4 acetylation, and lack of H3-K4 methylation occurred in the absence of DNA methylation. We demonstrate that DNA methylation within the transgene serves as an intrinsic epigenetic memory to prevent transgene reactivation. We also show that delayed DNA replication, combined with the lack of histone H3 and H4 acetylation and with H3-K9 methylation, are not sufficient to confer epigenetic memory in this system.

## Results

### Experimental System

**Figure 1:**
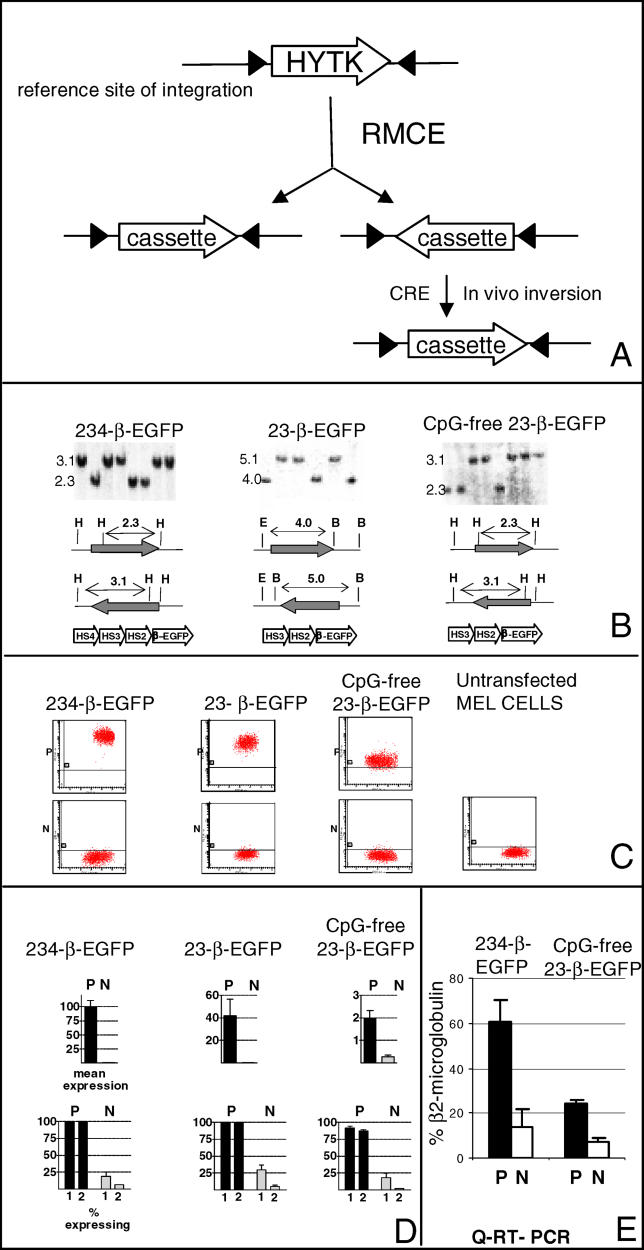
DNA Methylation Is Not Necessary for Silencing (A) Experimental system: reference integration sites are created by insertion in MEL cells of the HYTK gene flanked by two inverted L1Lox sites (black triangles). The HYTK gene can then be exchanged for any other cassettes by transfection of a Cre expression plasmid and of plasmids containing the cassette of interest. Clones with the cassettes in both orientations are obtained. Inserted cassettes can be inverted in vivo by re-expression of Cre. (B) Southern blots demonstrating insertion of cassettes 234-β-EGFP, 23-β-EGFP, and CpG-free 23-β-EGFP. For each cassette, multiple clones in each orientation are shown. (C) FACS analysis of typical clones in the P and the N orientations performed 6 wk post-integration. *x*-axis, forward-scatter; *y*-axis, EGFP fluorescence. All three cassettes are rapidly silenced in the N orientation, demonstrating that DNA methylation is not necessary for silencing at the RL4 locus. (D) Quantitative analysis of expression. Top histograms: Plots of normalized mean EGFP fluorescence (± SD) of three to five clones analyzed 2-mo post-integration in the P and N orientations. EGFP fluorescence of all cassettes is normalized to expression of cassette 234-β-EGFP in the P orientation at RL4. The CpG-free cassette in the P orientation is expressed at about 5% of the efficiency of the CpG-containing 23-β-EGFP cassette and 2% of the efficiency of the 234-β-EGFP cassette. Bottom histograms, percent of cells expressing EGFP, 1- and 2-mo post-integration. The cut-off between expressing and non-expressing cells is placed using untransfected MEL cells as controls. (E) Quantitative RT-PCR analysis of CpG-free cassette 23-β-EGFP and CpG-containing cassette 234-β-EGFP at RL4. Results are normalized to levels of β-2-micro-globulin. At the RNA level, the difference in expression between the CpG-containing and CpG-free cassettes is less than 3-fold. N, non-permissive orientation; P, permissive orientation.

We previously developed an experimental system to study expression and silencing of globin transgenes in mouse erythroleukemia cells (MEL). This system is based on the use of recombinase-mediated cassette exchange (RMCE), a method that utilizes the Cre recombinase to integrate transgenes at predefined chromosomal sites at high efficiency [25,26]. The principle of our experimental system is to create in the genome reference integration sites that can be re-used to study transgenes in a defined chromatin context (Figure 1A). To study silencing of globin reporter transgenes, we thus created in MEL cells three reference sites of integration (termed RL4, RL5, and RL6) by selection of clones containing single copies of a randomly integrated hygromycin-thymidine kinase fusion selectable marker (HYTK) flanked by two inverted lox sites. Using RMCE, we then replaced the *HYTK* gene with a series of reporter cassettes driven by various globin regulatory elements [27,28].

A particularity of our RMCE system is that insertion of the reporter cassettes at sites RL4, RL5, and RL6 occurs in both orientations because the Lox sites flanking the HYTK reporter are inverted [26]. This particularity turned out to be critical because analysis of these reporters revealed that expression depends on *the orientation* of the cassette relative to the flanking sequences at all three reference sites. For instance, expression of cassette 234-β-EGFP, which contains DnaseI hypersensitive sites 2, 3, and 4 of the human β-globin locus control region (LCR) and the human β-globin promoter driving the enhanced-green fluorescent protein (EGFP) reporter, is high when the cassette is integrated in one orientation at site RL4, but is rapidly silenced when the cassette is integrated in the opposite orientation. We term the orientation, in which the cassette expresses, the permissive orientation, the other, the non-permissive orientation. A similar phenomenon can be observed at site RL6, demonstrating that this silencing process is not idiosyncratic for one site of integration [27].

Because silencing at site RL4 is faster than at RL6 (6 wk versus 12 wk), we focused on this site to try to understand the mechanisms of initiation and maintenance of repressed chromatin structure, and in particular, the role of DNA methylation.

We previously reported that silencing at RL4 is associated with DNA methylation of the EGFP coding sequence, the promoter, and HS2 of the LCR [27]. In a separate study, we also reported that globin transgenes, pre-methylated in vitro at all their CpGs and subsequently inserted by RMCE at sites RL5 and RL6, were silenced regardless of their orientations. These studies demonstrated that DNA methylation was sufficient to cause silencing in MEL cells [29]. Similarly, Dalle et al. [30] have recently reported that silencing of reporter genes in mice and cell culture were dependent on CpG dinucleotides.

In the present study, we have created a cassette that cannot be methylated because it is totally devoid of CpG dinucleotides, and we have used this cassette to determine if methylation is necessary for silencing.

The 4,866 bases CpG-free 23-β-EGFP cassette was created by mutating 92 CpG dinucleotides in hypersensitive sites 2 and 3 of the β-globin LCR, the β-globin promoter, the coding sequence of the EGFP gene, and an SV-40-derived poly-adenylation signal. HS4 was deleted from the original cassette to minimize the efforts involved in mutating the cassette. To control for the effect of this deletion, we have also produced a CpG-containing 23-β-EGFP cassette.

Transient transfection experiments in MEL cells demonstrated that the CpG-free cassette was functional and produced the EGFP protein, albeit at a lower level than a CpG-containing 23-β-EGFP cassette (unpublished data).

To determine if the absence of CpG dinucleotides would prevent silencing, the CpG-free 23-β-EGFP cassette and two different CpG-containing control cassettes were inserted at integration site RL4 by RMCE (Figure 1B). Multiple clones with the cassette in either orientation were identified by Southern blot hybridization, and expression was then monitored by fluorescence-activated cell sorter (FACS) for several months.

### CpG Methylation Is Not Necessary for Silencing

As expected, the control CpG-containing cassette 234-β-EGFP was expressed at high levels when it was integrated in the permissive orientation but was completely silenced 4–6 wk after integration in the non-permissive orientation (Figure 1B, 1C, and 1D). The control CpG-containing 23-β-EGFP cassette behaved similarly: It was stably expressed (at about 40% of the level of cassette 234-β-EGFP) when integrated in the permissive orientation, and was completely silenced 4–6 wk after integration in the non-permissive orientation (Figure 1B, 1C, and 1D), demonstrating that deletion of HS4 has no effect on silencing.

Interestingly, the CpG-free 23-β-EGFP cassette behaved as the control cassettes. It was stably expressed when integrated in the permissive orientation and silenced within 6 wk in the non-permissive orientation. This result clearly demonstrates that at this site of integration, CpG methylation of the β-globin promoter and the LCR was not necessary for silencing.

The flow cytometry results also revealed that in the permissive orientation, the CpG-free cassette was expressed at only 5%–10% of the level of the control 23-β-EGFP cassette. To determine whether this was due to sub-optimal codon usage in the CpG-free EGFP coding sequence or to a decreased transcriptional activity of the CpG-free mini-LCR and promoter, we performed quantitative RT-PCR on total RNAs extracted from cells containing the CpG-containing and CpG-free cassettes. This revealed that at the RNA level, expression of the CpG-free 23-β-EGFP cassette was only two to three times lower than the control 234-β-EGFP (Figure 1E). We therefore conclude that the major cause of the low level of expression of the CpG-free cassette in the permissive orientation was post-transcriptional.

Because variations of gene expression can stem from a variety of biochemical mechanisms, it was essential to establish whether the silencing observed with the CpG-free cassette at the non-permissive orientation shared some or all of the characteristics of gene silencing that we previously observed with the CpG-containing cassettes. Two important features of the orientation-dependent silencing are the replication delay and the appearance of histone post-translational modifications associated with condensed chromatin. To determine which histone modifications are associated with silencing of the CpG-free transgenes, we used the chromatin immuno-precipitation assay (ChIP) to measure the levels of acetylation and methylation of histones H3 and H4 in chromatin derived from cells containing the CpG-free 23-β-EGFP cassette in either orientation. We used primers for the EGFP coding sequence and the β-globin promoter, both contained within the transgene, to assess the abundance of transgene sequences in chromatin enriched for specific histone modifications. As controls, we also used primers for the mouse β-major globin and amylase genes which are known to be associated with condensed and decondensed chromatin in these cells, respectively.

**Figure 2:**
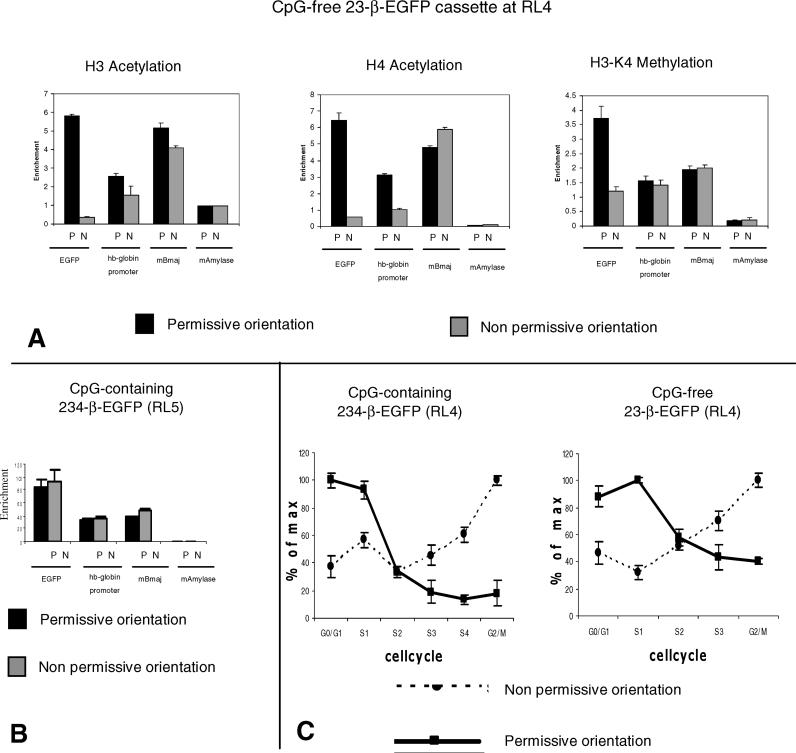
CpG-Free Silencing Is Associated with Lack of Acetylation of H3 and H4, Lack of Methylation of H3-K4, and Replication in Late S Phase (A) Histone modification analysis by ChIP assays. The histograms summarize the results of quantitative PCR measurements of abundance of the EGFP coding sequence, the hβ-globin promoter, the mouse β-major promoter region (mBmaj), and a fragment of the mouse amylase coding sequence (mAmylase) in chromatin from cells containing cassette 23-β-EGFP at RL4 precipitated with polyclonal antibodies against acetylated H3-tails (left), acetylated H4-tails (middle), and methylated H3-K4-tails. The enrichment of specific sequences in modified chromatin compared to total chromatin was calculated as described in Materials and Methods. Both the EGFP coding sequence and the β-globin promoter of the CpG-free 23-β-EGFP cassette are enriched when acetylated on H3 and H4 and H3-K4 methylation and when they are in the P orientation, but not when they are in the N orientation. (B) ChIP analysis of the CpG-containing cassette inserted at RL5 in the N or P orientation. The two orientations are similar, and, as at RL4, the enrichment is higher for the EGFP coding sequences than for the promoter. (C) Timing of replication analysis: The histograms summarize the results of quantitative PCR measurements of the abundance of the CpG-free and CpG-containing EGFP coding sequence in FACS-separated, BrdU-immunoprecipitated newly replicated DNA fragments from cells containing the CpG-containing (left) or the CpG-free (right) LCR-β-EGFP cassettes. Both cassettes replicate early when they are in the P orientation and late when they are in the N orientation. Controls were as in Figure 3. N, non-permissive orientation; P, permissive orientation.

When the CpG-free cassette was integrated in the permissive orientation at RL4, sequences from the human β-globin promoter and within the coding sequence of EGFP were abundant in chromatin enriched for acetylated histones H3 and H4 and for histone H3 methylated at K4 (Figure 2A). By contrast, when the CpG-free cassette was in the non-permissive orientation, the EGFP coding sequence (and to a lesser extent the β-globin promoter) were not enriched in chromatin containing acetylated H3 and H4 and methylated H3-K4. These results are similar to our previous observations on the CpG-containing cassettes [24] and demonstrate that the changes in histone modifications associated with silencing at RL4 occur independently from DNA methylation.

**Figure 3:**
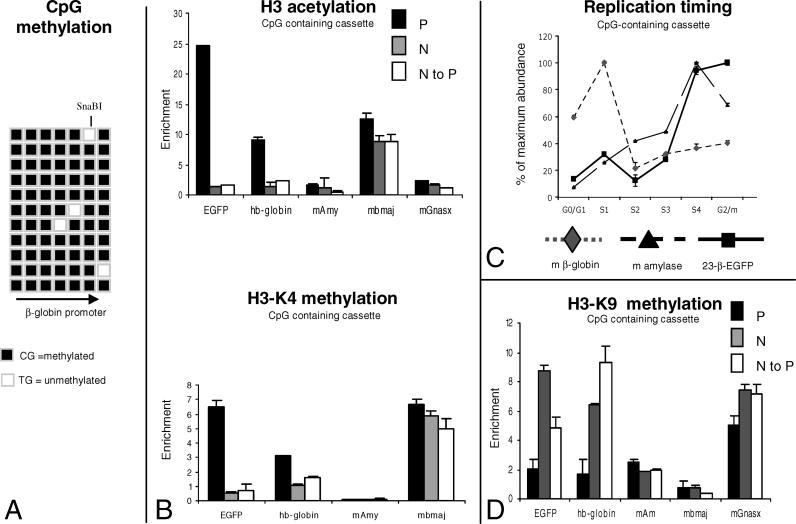
Analysis of the Chromatin Structure of the CpG-Containing Cassette (A) Bisulfite sequencing analysis was performed on cells with CpG-containing cassette 234-β-EGFP at RL4 after N to P inversion. There were 11 molecules sequenced. Most of the CpGs were methylated (see text). (B) Histograms illustrating H3 acetylation and H3-K4 methylation results on the CpG-containing cassettes. The analysis was performed as in Figure 2. Mouse β-major globin (mBmaj) is a positive control for H3 and H4 acetylation and a negative control for H3-K9 tri-methylation. The α subunit of the guanine nucleotide-binding protein (mGnasx) and the mouse amylase genes are a positive control for H3-K9 tri-methylation and negative controls for H3 acetylation. The CpG-containing cassette is enriched in H3 acetylation and H3-K4 methylation in the P orientation but poor in acetylated H3 and H3-K4 methylation. After N to P inversion, the cassette remains poor in H3 acetylation and H3-K4 methylation. (C) Plots illustrating replication timing analysis of the CpG-containing cassette after N to P inversion. The analysis was performed as in Figure 2. Mouse β-major globin and amylase are early and late replicating controls. After N to P inversion, the CpG-containing cassette replicate late in S phase. N, non-permissive orientation; P, permissive orientation. (D) Histograms illustrating H3-K9 tri-methylation results on the CpG-containing cassettes. The analysis was performed as in Figure 2. The controls are as in Figure 3B. The enrichment is low when the cassette is in the P orientation and high in the N orientation. The enrichment remains high after N to P inversion.

These studies also revealed that the enrichment in acetylated histone H3 and methylated H3-K4 was higher on the coding sequence than on the promoter. To determine if this was specific to the RL4 site of integration, we performed ChIP experiments on MEL cells with the CpG-containing cassette integrated at RL5, a site of integration similar to RL4, but where silencing does not occur. These experiments revealed that, as at RL4, the enrichment was higher in the coding sequence than in the promoter region (Figure 2B), perhaps indicating that the promoter region is partly depleted in nucleosomes.

We then assessed the timing of replication of the CpG-free cassette by measuring the abundance of sequences from the CpG-free cassette in BrdU-labeled, newly replicated DNA from asynchronous cells fractionated by FACS according to their positions in the cell cycle. As controls, we determined the timing of replication of sequences in the murine β-globin and amylase loci. Figure 2C shows that sequences from the CpG-free cassette were abundant in the early S-phase fractions when the cassette was in the permissive orientation and abundant in the late S-phase fraction when the cassette was in the non-permissive orientation. This demonstrates that the changes in replication timing associated with silencing at RL4 occur independently from DNA methylation. Again, these results are similar to those obtained with the control CpG-containing cassettes.

### CpG Methylation Confers Epigenetic Memory

Because transgenes integrated at RL4 are flanked by inverted Lox sites, they can be inverted in vivo by re-expression of Cre in the cells (Figure 1A). We have taken advantage of this property to examine the maintenance of the epigenetic marks of the open and closed chromatin structures that form at RL4. We previously reported that inversion of a CpG-containing cassette from the permissive to the non-permissive orientation leads to its rapid silencing, but that inversion of a CpG-containing silenced cassette from the non-permissive to the permissive orientation does not lead to its full reactivation [31]. Instead, expression of the inverted cassette occurred at low levels in a small number of cells. This demonstrated that silencing leaves a permanent epigenetic mark on the transgene, since the inverted cassette retained a memory of having been silenced. We termed this memory phenomenon “somatic imprinting.” In the same report, we also observed that the somatically imprinted cassettes (inverted from the non-permissive to the permissive orientation) retained their DNA methylation at the promoter and in the coding sequence of EGFP using methylation-sensitive restriction nucleases [32]. To extend these results to the individual CpG level, we have performed a DNA methylation analysis by bisulfite sequencing on clones with a non-permissive to permissive inversion that had been in culture for more than 3 mo. As shown in Figure 3A, this analysis revealed that the seven CpG dinucleotides analyzed were 90%–100% methylated; demonstrating that methylation of the β-globin promoter was very stable in the inverted cassette.

To further investigate the physical support of this epigenetic memory, we performed ChIP on the inverted CpG-containing cassette using primers located in the β-globin promoter and in the coding sequence of EGFP. This revealed that after non-permissive to permissive inversion, the 234-β-EGFP cassette is poor in histone H3 acetylation and H3-K4 methylation (Figure 3B). As for the CpG-free cassette, the enrichments observed were generally higher for the coding sequence than for the promoter.

Since previous analyses had demonstrated striking differences of replication timing of the CpG-containing cassette in the non-permissive and permissive orientation, we determined the timing of replication of the CpG-containing cassette after non-permissive to permissive inversion (Figure 3C). This revealed that the non-permissive- to permissive-inverted CpG-containing cassette replicated late in S phase, and therefore the timing of replication was not changed by the inversion.

Since tri-methylation of Lys 9 of histone H3 has been associated with epigenetic memory through recruitment of HP1 [10], we also performed ChIP analysis using antibodies specific for this histone modification. As described in Figure 3D, when the CpG-containing cassette was inserted in the permissive orientation it was not enriched in tri-methyl-H3-K9 histones. In contrast, when the same cassette was inserted in the non-permissive orientation and therefore silenced, it was highly enriched for this modification. As expected, control PCR revealed that the mouse β-major promoter (expressed) was poor in tri-methyl-H3-K9 while the mouse major satellite and the α subunit of the guanine nucleotide-binding protein (*mGnasx,* an imprinted gene) were highly enriched in tri-methyl-H3-K9. We conclude from these experiments that the silenced chromatin that forms when cassette 234-β-EGFP is inserted in the non-permissive orientation at RL4 is associated with tri-methylation of H3-K9. ChIP analysis after inversion of the silenced CpG-containing cassette back toward the permissive orientation revealed that tri-methylation of H3 was retained.

Together, these experiments revealed that the inverted CpG-containing cassette had retained the epigenetic marks that it had acquired on its promoters and coding sequences when it was silenced in the non-permissive orientation. Based on these results we hypothesized that the epigenetic memory that imprints the cassette after its inversion could be CpG methylation, a histone post-translational modification, or replication in late S phase.

**Figure 4:**
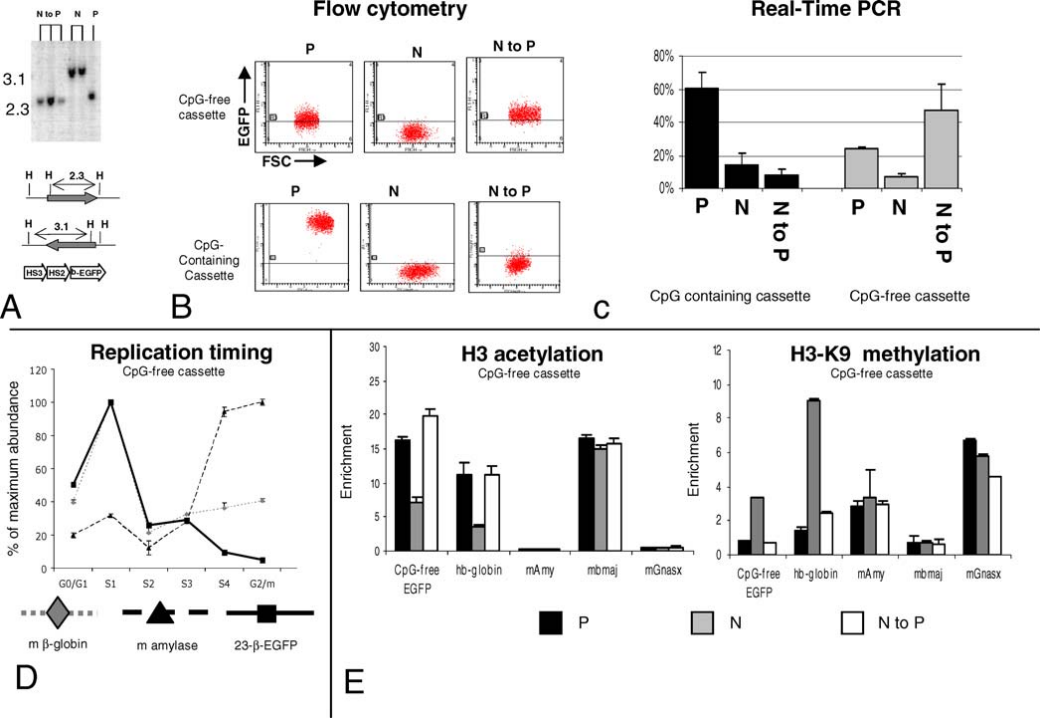
Inversion of the CpG-Free Cassette and Analysis of Chromatin Structure of the Inverted Cassette (A) Southern blots demonstrating inversion of the 23-β-EGFP cassette from the N to the P orientation. N to P lanes, Hind III digested genomic DNA from three N to P inverted sub-clones isolated after transfection of cells with cassette CpG-free 23-β-EGFP in the N orientation with a Cre recombinase expression plasmid. N and P, control sub-clones. (B) FACS analysis of representative clones with the CpG-free and CpG-containing cassettes in the P or N orientation or after N to P inversion. *x*-axis, forward scatter; *y*-axis, EGFP fluorescence. N to P inversion leads to the reactivation of the CpG-free but not the CpG-containing cassette. (C) Quantitative RT-PCR analysis of EGFP expression on total RNA extracted from two to five sub-clones with the CpG-containing or the CpG-free cassette in the P or N orientation and after N to P in vivo inversion. Results are expressed as percent of β-2-micro-globulin expression (100 × number of EGFP transcripts/number of B2M transcripts). This analysis confirms the FACS data and demonstrates that silencing and reactivation of the CpG-free cassette occurs primarily at the transcriptional level. (D) Plots illustrating replication timing analysis of the CpG-free cassette after N to P inversion. The graphs and controls are as in Figure 3C. After N to P inversion, the CpG-free cassette replicates early in S phase. (E) ChIP analysis of the CpG-free 23-β-EGFP cassette. The graphs and controls are as in Figure 3B. The results show that the CpG-free cassette is enriched in H3 acetylation and poor in H3-K9 tri-methylation in the P orientation, but that it is poor in H3 acetylation and rich in H3-K9 tri-methylation in the N orientation. After N to P inversion, the CpG-free cassette becomes enriched in H3 acetylation but poor in H3-K9 tri-methylation. N, non-permissive orientation; P, permissive orientation.

To differentiate between these possibilities, we have inverted the CpG-free 23-β-EGFP cassette that had been inserted at RL4 in the non-permissive orientation for 3 mo and was therefore completely silenced. Multiple individually isolated clones containing the CpG-free 23-β-EGFP were transfected with a Cre expression plasmid and 1 mo after the transfection, sub-clones with the inversion were identified by Southern blots (Figure 4A). Expression levels were then determined by FACS and compared with CpG-containing and CpG-free controls (Figure 4B). In sharp contrast with the results obtained with the CpG-containing cassette, this analysis revealed that inversion of the CpG-free cassette from the non-permissive to the permissive orientation led to its rapid reactivation. Therefore, in the absence of CpG methylation, the cassette loses its epigenetic memory. Quantitative RT-PCR analysis on total RNA confirmed these results (Figure 4C) and demonstrated that the silencing occurs primarily at the transcriptional level. We conclude from these experiments that DNA methylation contributes to the permanent mark that confers memory to these cassettes.

Replication timing analyses revealed that the timing of replication of the CpG-free cassette that had been inverted from the non-permissive to the permissive orientation had reverted to early S phase (Figure 4D). Therefore, the replication delay that persisted in the inverted CpG-containing cassette did not persist in the absence of CpG methylation. ChIP analyses revealed that sequences derived from the inverted CpG-free cassette were enriched in chromatin-containing acetylated histone H3 (Figure 4E), again contrasting with the results obtained with the inverted CpG-containing cassette, which maintained the deacetylated chromatin state. We therefore conclude that CpG methylation is required to maintain the delayed replication and the deacetylated histone phenotypes when the cassettes are inverted toward the permissive orientation.

ChIP analysis revealed that insertion of the CpG-free cassette in the permissive orientation is associated with low levels of enrichment for tri-methyl-H3-K9, and that insertion in the non-permissive orientation is associated with high levels of enrichment for tri-methyl-H3-K9 (Figure 4E), suggesting that establishment of this histone modification does not require DNA methylation. Importantly, inversion of the CpG-free cassette from the non-permissive to the permissive orientation revealed a complete loss of enrichment for this modification. As for histone acetylation, we therefore conclude that CpG methylation is required to maintain the tri-methylation of histone when the cassettes are inverted toward the permissive orientation.

Similar experiments using an anti-tri-methyl H3-K27 antibody did not reveal any enrichment for this modification in these cassettes in either orientations or in several control DNA segments, suggesting that this modification is not commonly present in single-copy active or inactive genes in MEL cells, and that it does not seem to play a role in silencing of these transgenes (unpublished data).

### Cre Recombination Per Se Does Not Alter Expression

**Figure 5:**
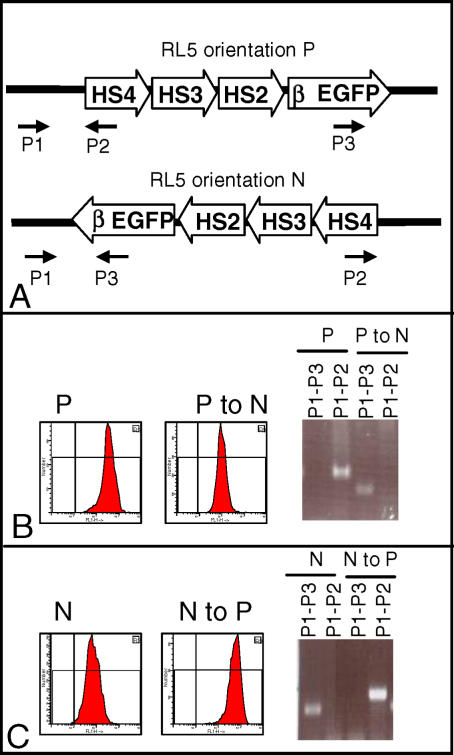
Cre Recombination Per Se Does Not Lead to Silencing Cells containing cassette 234-β-EGFP at RL5 were transfected with the Cre recombinase, and inverted sub-clones were identified using primer pairs p1/p2 (specific for the P orientation) and p1/p3 (specific for the N orientation). (A) Location of the primers used to identify the inverted clones. (B) P to N inversion: The histogram on the left illustrates expression profile of a clone with insertion in the P orientation before inversion. The histogram on the right illustrates expression 8 wk post-inversion. The ethidium bromide stain illustrates the PCR analysis demonstrating the inversion. (C) Same as (B) but for the N to P inversion. This analysis clearly demonstrates that Cre recombination per se does not lead to silencing (see text). As reported previously, levels of expression in the N orientation at RL5 are lower than in the P orientation [32]. N, non-permissive orientation; P, permissive orientation.

One possible interpretation of some of these results was that the changes in chromatin structure that we observe after inversion are caused by the recombination process itself, since recombination involves the creation of double-stranded breaks that might alter the topological conformation of the chromatin or recruit repair proteins that would in turn recruit chromatin- modifying proteins. Since the CpG-containing 234-β-EGFP cassette inverted from the non-permissive to the permissive orientation at RL4 retains its chromatin structure, we can eliminate the possibility that the inactive chromatin structure is altered by the recombination process when DNA methylation is present. To test the possibility that the active chromatin structure is altered by the recombination process, we inverted cassette 234-β-EGFP after it was inserted at RL5, a site at which expression occurs in both orientations (Figure 5) (albeit about 50% lower in one of the two orientations). These experiments revealed that inversions at RL5, regardless of the starting orientation, did not lead to silencing; demonstrating that the recombination process itself does not lead to the collapse of the active chromatin structure. These experiments do not exclude that the recombination event could have subtler effects on the chromatin structure.

## Discussion

Gautsch and Wilson [33], more than 20 years ago, suggested that DNA methylation might be necessary for maintenance of transgene repression but not for its initiation in early embryos. Since then, several other chromatin features have been associated with gene repression, and we now know that transgene silencing is generally associated with CpG methylation, delayed replication, low levels of H3-K4 methylation, H3 and H4 acetylation, and high levels of tri-methyl-H3-K9 or K27 [34]. While these associations have been clearly demonstrated, the relationships between these chromatin marks are complex and difficult to decompose. Knockout and over-expression studies have provided profound insights regarding the roles of DNA methylation and some of the histone modifications, but redundancies can mask some functions. In addition, complete elimination of DNA methylation or of histone modifications have cell-wide consequences, complicating interpretation, since the observed phenotypes are potentially indirect. In this study, we have taken advantage of the RMCE method and of a CpG-free cassette to dissect the role of CpG methylation in relation to other known epigenetic modifiers in the control of expression of a single-copy gene using silencing at RL4 as a model. The mechanism of orientation-dependent silencing at this site of integration remains incompletely understood. However, we have recently provided evidence that the triggering event for silencing might be transcriptional interference induced by the presence of the β-globin mini-LCR [35].

We report here that at site RL4 transcriptional silencing, delayed replication, enrichment in tri-methyl-H3-K9, and lack of histone acetylation and H3-K4 methylation occurs independently of CpG methylation.

In most experiments, enrichment in the ChIP experiments was higher for the EGFP coding sequence than for the promoter: whether this reflects the fact that the promoter is depleted in nucleosomes is unclear. In any case, these experiments show that CpG methylation is not necessary for the initiation and maintenance of the heterochromatic structure that forms at RL4, and that histone modifications and delayed replication are sufficient to prevent expression in the non-permissive orientation at this site of integration. A previous report has shown that DNA methylation is sufficient to initiate transgene silencing [29] without requiring delayed replication. More recently, Mutzkov et al. [34] have suggested that methylation of H3-K9 was not the primary causative event for silencing of transgenes in chicken erythroid cells.

Together these data suggest that a silent heterochromatic structure can be initiated either by CpG methylation or by histone modifications and delayed replication, and that these features can be acquired in more than one order, maybe because of the multiple interactions that are known to exist between Dnmt and histone-modifying enzymes. This raises the question of whether these chromatin features have unique functions. As discussed below, the cassette inversion experiments suggest that CpG methylation differs from the other chromatin marks studied above because it confers epigenetic memory.

### Epigenetic Memory

The chromatin structure of silenced transgenes is very stable at RL4: clones containing silenced cassettes in the non-permissive orientation can be kept in culture for more that 1 y and will never reactivate (unpublished data). The epigenetic information is therefore faithfully replicated through hundreds of cell divisions suggesting the existence of an epigenetic memory. This epigenetic memory can theoretically result from two types of mechanisms: the silent chromatin structure could be maintained through cell division simply because the factors required for its establishment remain present in the cell at all times. Alternatively, after initiation of silencing, the chromatin might acquire some inherent characteristics that allow the propagation of the epigenetic information even when the initiating factors are no longer present. We propose that there are two types of epigenetic memory. The first, termed apparent memory would require the continued presence of an initiating signal. The second, termed intrinsic memory would persist even in the absence of the initiating signal. We also propose that both intrinsic and apparent epigenetic memories have important but different biological roles in mammalian cells. Intrinsic epigenetic memory could be particularly useful to permanently silence retroviruses and transposons throughout development and differentiation, or oncogenes in cell types where they can cause transformation. Apparent epigenetic memory could be more appropriate to temporarily silence genes that need to be re-expressed at a later time during differentiation or in response to induction by environmental agents.

We have observed here that the effects of in vivo inversion of a silent heterochromatic cassette depended on the presence of CpG dinucleotides: If the cassette contained CpG dinucleotides, the chromatin remained condensed and expression continued to be silenced. If the cassette did not contain CpGs, the chromatin converted to a decondensed configuration and expression was reactivated.

These experiments unambiguously demonstrate that CpG methylation is necessary for the persistence of the epigenetic information acquired in the non-permissive orientation. We conclude that DNA methylation confers intrinsic epigenetic memory since the silent-state persists after a major perturbation of the chromatin structure that eliminates the initiating silencing event.

In the absence of CpG methylation, all of the marks of silent chromatin that we tested were erased. We have therefore failed to detect any evidence that histone modifications or delayed replication confer an intrinsic epigenetic memory, independently of DNA methylation. This result can be interpreted either by the hypothesis that these chromatin marks are not self-replicating and are unable to confer intrinsic epigenetic memory or by the hypothesis that except for DNA methylation, a covalent modification, all of the chromatin marks tested were erased by the Cre-mediated inversion event itself. The latter hypothesis is not supported by the experiments in Figure 5 which show that at locus RL5, Cre-mediated inversions had no effects on expression; or by reports by Ghirlando et al. [36] and Boeger et al. [37] that in vivo excision of chromatin fragments with restriction nucleases or with the Cre recombinase do not dramatically perturb the chromatin structure.

In any case, we have demonstrated that among the five chromatin characteristics tested, methylation of CpG dinucleotides is unique in its ability to confer an intrinsic epigenetic memory that persists after a DNA rearrangement. It remains to be determined if in mammalian cells, histone-code based, intrinsic memory systems independent of DNA methylation exist, and if they do, whether they are active at all developmental times or restricted to early development. Conferring intrinsic epigenetic memory might be a critical, non-redundant function of DNA methylation that plays an important role in the protection of genome integrity and accounts for its conservation in many organisms.

## Materials and Methods

### Plasmids.

The CpG-free EGFP coding sequence was constructed by mixing five overlapping 200 bases synthetic oligonucleotides in a single PCR reaction, resulting in the self-assembly of the CpG-free cDNA. Sub-cloning and sequencing of this CpG-free cDNA revealed the presence of five unwanted substitutions that were corrected by conventional recombinant PCR. The CpG-free β-globin promoter was then added to the CpG-free cDNA by a three-way ligation of PCR products generated with long primers that contained mutations that eliminated all CpG dinucleotides from the promoter. Finally the LCR part of the construct was added as a 1.1-kb synthetic fragment synthesized by a commercial supplier. The entire plasmid was sequenced. A total of 92 CpG dinucleotides were eliminated: four CpGs in HS3, 11 CpGs in HS2, and three CpGs in the promoter were replaced by nucleotides found in the homologous sequences of primates or mice. In the coding sequences, 61 CpGs were replaced by nucleotides that yield synonymous codons without dramatically altering codon usage [38]. Finally, 13 CpGs that were located in remnants of multiple cloning sites between the various cis-elements of the cassette were deleted. Plasmid 234-β-EGFP has been described previously [31]. Plasmid 23-β-EGFP is identical to 234-β-EGFP except for the deletion of HS4. Details of the construction and sequences of all plasmids are available on request. Tissue cultures were performed as in [27,31].

### RMCE and cassette inversions.

These experiments were performed as previously described [26,31]. Briefly, 2–4 million MEL cells containing the HYTK cassette and under-hygromycin selection were electroporated with 200 μg of exchange cassette and 50 μg of Cre expression plasmid and selected in gancyclovir for 10 d. Sub-clones were then picked and analyzed by Southern blots. At least three sub-clones with the cassettes in each orientation were identified and subsequently analyzed. Inversions were performed, either by electroporating 2–4 million cells with 50 μg of Cre expression plasmid or by transfection of 100,000 cells with 1μg of Cre expression plasmid using Fugene (Stratagene, La Jolla, California, United States). All Southern blots were probed with the EGFP coding sequence. The sequence of primers p1, p2, and p3 is available on request.

### Expression analysis.

FACS analyses were performed as described earlier [27]. Quantitative RT-PCR on total RNAs were performed using the Light-Cycler (Roche, Basel, Switzerland) and the Qiagen (Valencia, California, United States) Quantitech kit according to the manufacturer instructions. The primers and probes used are available on request. All quantifications were performed at least in duplicate using at least three independently derived clones. To estimate the number of molecules present in each sample, standard curves were created using purified PCR products. Expression results were normalized to the number of β-2-microglobulin molecules present in each sample.

### Replication timing analyses.

These experiments were performed as described previously [24]. Briefly, asynchronously growing cells were labeled with the thymidine analog BrdU for 90 min and fractionated based on DNA content after Hoechst 33342 labeling using a Vantage FACS. Newly replicated, BrdU-substituted DNA was isolated by immunoprecipitation. After two rounds of immunoprecipitation and DNA isolation, mitochondrial DNA sequences (mMT primers) were used to verify that each fraction contained similar quantities of amplifiable DNA strands. Samples containing 8 ng of DNA were analyzed by real-time PCR on an ABI 7900 (Columbia, Maryland, United States). The sequences of primers and probes are available on request. The abundance of an amplified sequence was calculated using genomic DNA standards with a standard curve ranging from 0.005 ng–50 ng per reaction. Each measurement was performed in triplicate. The relative abundance for each probe/primer combination was calculated as the ratio of the number of molecules amplified from a specific cell-cycle fraction to the number of molecules amplified from the cell-cycle fraction where amplification was maximal. Each experiment was repeated twice on one or two independently derived clones.

### ChIP assays.

These assays were carried out as described previously [24]. MEL cells were fixed for 5 min at room temperature by the addition of 1% formaldehyde to the growth medium. After a series of washes, chromatin was shredded by sonication and subjected to immunoprecipitation with antibodies against modified histones. Antibodies used included anti-acetyl-histone H3 (06–599), anti-acetyl-histone H4 (06–866) and anti-dimethyl-histone H3 (Lys 4) (07–030), anti-tri-methyl-histone H3 (Lys 9) (07–442), and anti-trimethyl-histone H3 (Lys 27) (05–851), all from Upstate Biotechnology (Lake Placid, New York, United States). DNA was released by heating followed by treatment with RNAse and proteinase K. DNA concentrations of the samples were determined by Pico green fluorescence (Molecular Probes, Eugene, Oregon, United States). Real-time PCR was used to amplify same amount of ChIP-enriched DNA and input genomic DNA. The enrichment in the abundance of the transgenes or host genes in acetylated chromatin was calculated as the ratio between the abundance of the amplified sequences in immunoprecipitated DNA and the abundance of the amplified sequences in total genomic DNA. Each experiment was repeated twice with independently derived clones. The sequences of primers and probes are available on request. Because of sequence differences between the CpG-containing and CpG-free cassettes, the sequences of the primers and probes used to assay these two transgenes were not identical. We therefore limited our interpretations of the difference between the CpG-free and CpG-containing cassette to ratios between enrichment values rather than to the absolute values.

### Real-time PCR primers and probes.

All real-time PCR reactions for the replication timing and ChIP analysis were performed on an ABI Prism 7900HT SDS. The sequences of all the primers and probes used in this study are available on request.

### Methylation analysis.

Bisulfite conversion was performed as described previously [31,32].

## Abbreviations

ChIP: chromatin immuno-preciptation
Dnmt: DNA methyltransferase
EGFP: enhanced-green fluorescent protein
FACS: fluorescence-activated cell sorter
HYTK: hygromycin-thymidine kinase
LCR: locus control region
MEL: mouse erythroleukemia
RMCE: recombinase-mediated cassette exchange
